# Dielectric Properties and Defect Chemistry of Tb/Ho-Co-Doped BaTiO_3_ Ceramics

**DOI:** 10.3390/ma18122914

**Published:** 2025-06-19

**Authors:** Junwei Liu, Xin Wei, Qiaoli Liu, Yupei Ran, Guoqi Xu, Qi Liu

**Affiliations:** 1 School of Ceramics, Pingdingshan University, Pingdingshan 467000, China; phys137@foxmail.com (J.L.);; 2 Yaoshan Laboratory, Pingdingshan 467000, China; 3 Huangmei Longyuan Gypsum Co., Ltd., Huangmei 435500, China; wx_0915kkx@163.com

**Keywords:** Tb/Ho-co-doped BaTiO_3_, dielectric properties, point defect, site occupancy

## Abstract

Co-doping at Ba and Ti sites with double rare-earth elements has proven an effective strategy for enhancing the dielectric properties of BaTiO_3_ ceramics. Among intermediate-sized rare-earth ions, Tb and Ho exhibit amphoteric behavior, occupying both Ba and Ti sites. Investigating the site occupation, defect chemistry, and dielectric effects of Tb and Ho in BaTiO_3_ is therefore valuable. In this work, Tb/Ho-co-doped BaTiO_3_ ceramics with the composition (Ba_1−*x*_Tb*_x_*)(Ti_1−*x*_Ho*_x_*)O_3_ (*x* = 0.01~0.10) were fabricated at 1400 °C via solid-state reaction, and their solid solubility and crystal structures are confirmed. Microstructure, dielectric properties, photoluminescence, and valence states of samples with a single phase were systematically studied. Both the lattice parameter *a* and unit cell volume increase with doping level. The ceramic with *x* = 0.02 meets the X5S dielectric specification. Ho and Tb ions both demonstrate amphoteric site occupancy: Ho exists solely as Ho^3+^ at both Ba and Ti sites, while Tb exhibits mixed valence states as Ba-site Tb^3+^ and Ti-site Tb^4+^. As the doping content increases, the concentration of Tb^4+^ at Ti sites decreases, and the quantity of Ba-site Ho^3+^ ions initially increases to a maximum before decreasing. Defect compensation mechanisms within the samples are also discussed.

## 1. Introduction

High-performance dielectric ceramics with perovskite structure (ABO_3_) have attracted huge attention for their unique properties. For example, BaTiO_3_ [1,2], SrTiO_3_ [3], and (K, Na)NbO_3_ [4] have been widely used in high-*k* dielectrics, piezoelectric devices and so on. Among these compounds, BaTiO_3_-based ceramics are widely used in multilayer ceramic capacitors (MLCCs) [5,6], positive temperature coefficient (PTC) thermistors [7,8], and other applications. Rare-earth elements are found to be crucial to the electrical properties of BaTiO_3_, and various doping strategies have been adopted to improve the performance of the above devices. Hu et al. prepared Bi- and Ho-co-doped BaTiO_3_ ceramics and studied the synergistic effects of Bi and Ho on the dielectric performance. By adjusting the doping concentration, the samples satisfied the X8R specification and exhibited good temperature coefficient of capacitance stability [9]. Lu et al. designed rare-earth element and Ca co-doped BaTiO_3_ with the formula of (Ba_1−*x*_RE*_x_*)(Ti_1−*x*/2_Ca*_x_*_/2_)O_3_ (RE = La, Tb, Dy) and studied their dielectric properties in detail, and the samples showed high dielectric performance and satisfied the X5R, X7R, or X8R specifications [10,11,12]. These studies demonstrate convincingly that rare-earth elements play an important role in tailoring performance for BaTiO_3_ ceramics. Apart from the above doping strategy, simultaneous doping at Ba and Ti sites with double rare-earth elements is also proved to be effective for improving the dielectric properties of BaTiO_3_ [13].

The site occupation of rare-earth ions in the BaTiO_3_ lattice depends on the ionic radii [14,15]. Large rare-earth ions, for example, La^3+^ and Sm^3+^, occupy exclusively the Ba site, whereas the small ions such as Yb^3+^ enter the Ti site. Using a double rare-earth doping strategy, the researchers could prepare dielectric ceramics with fine grains and low loss [16,17]. Ions with intermediate size, for example, Ho^3+^, Er^3+^, and Dy^3+^, exhibit an amphoteric behavior of occupying Ba sites as donors and Ti sites as acceptors [18,19].

Among the intermediate-size rare-earth ions, Ho is important for the densification and dielectric properties of BaTiO_3_ ceramics. Lu et al. studied Ho-doped BaTiO_3_ ceramics with a self-compensation mechanism, and they found that Ho-doped BaTiO_3_ ceramics have a high relative density and a low dielectric loss [20]. Tb also exhibits amphoteric behavior, i.e., Tb ions can occupy both Ba and Ti sites. However, unlike Ho, which keeps a stable trivalent state, Tb ions may exist in the BaTiO_3_ lattice as Tb^3+^ or Tb^4+^. Some authors proposed the possibility that Tb ions exist at Ti sites as Tb^3+^ [19,21,22], whereas Tsur et al. pointed out that Tb could be incorporated into Ti sites as Tb^4+^ during the sintering process [23]. Lu et al. proved that the self-compensation mode of Tb (${Tb}_{Ba}^{\cdot }-{Tb}_{Ti}^{\prime }$) does not exist in the case of Ba/Ti = 1 because Tb occupies Ba and Ti sites as Tb^3+^ and Tb^4+^, respectively [24]. The results of Lu et al. demonstrated that the self-adjustable amphoteric behavior of valence states of Tb ions is important for the lattice electroneutrality of the samples. Based on the above studies and discussion, it is meaningful to study the interaction of Tb and Ho in BaTiO_3_. In this work, Tb- and Ho-co-doped BaTiO_3_ samples are designed to explore the crystal structure, site occupancy, and dielectric properties.

## 2. Materials and Methods

(Ba_1−*x*_Tb*_x_*)(Ti_1−*x*_Ho*_x_*)O_3_ (*x* = 0.01~0.10) (abbreviated as BTTH) ceramics were prepared by the conventional solid-state reaction method. Starting reagents BaCO_3_ (99.4%, Sinopharm Chemical Regent Co., Ltd., Shanghai, China), TiO_2_ (99.5%, Yuejiang Chemical Co., Ltd., Shanghai, China), Tb_4_O_7_ (99.99%, Diyang Co., Ltd., Shanghai, China), and Ho_2_O_3_ (99.99%, Diyang Co., Ltd., Shanghai, China) were weighed according to their stoichiometric ratios. These raw material oxides were thoroughly ground in an agate mortar and calcined at 1100 °C for 5 h. The calcined powders were mixed with 12 wt% PVA and pressed into cylindrical pellets (12 mm in diameter) under 200 MPa. Finally, these pellets were sintered at 1400 °C for 12 h in an air conditioner.

The room-temperature crystal structures were determined by powder X-ray diffraction (XRD) using a diffractometer (DX-2700, Dandong, China) with Cu *Kα* radiation (1.540562 Å), and the measurement step and counting time were 0.02° and 3 s, respectively. The microstructures of the pellets were characterized by scanning electron microscopy (SEM, EVO MA10, Zeiss, Oberkochen, Germany). The Nano Measurer software (Version 1.02) was used to estimate the grain size distribution of SEM images. X-ray photoelectron spectroscopy (XPS) measurements were carried out on a Thermo ESCALAB 250Xi spectrometer (Waltham, MA, USA) with Al *Kα* radiation to study the valence states. The XPS curves were fitted by a mixed Gaussian-Lorentzian function, and a Shirley-type background subtraction was used. Photoluminescence (PL) spectroscopy was adopted to investigate the site occupancy of Ho^3+^ ions on a LabRAM XploRA Raman spectrometer (HORIBA France SAS, Longjumeau, France) with a 532 nm laser (Horiba Jobin Yvon). The pellets were polished on both sides, and then silver paste was painted as an electrode before dielectric measurement. The dielectric properties were performed on a broadband dielectric spectrometer (Concept 41, Novocontrol Technologies, Montabaur, Germany) in the temperature range of −75–200 °C with a heating rate of 2 °C/min. Room-temperature electron paramagnetic resonance (EPR) measurement was carried out with an X-band (≈9.85 GHz) spectrometer (A300, Bruker, Berlin, Germany).

## 3. Results and Discussion

### 3.1. Crystal Structure and Microstructure Characterization

**Figure 1:**
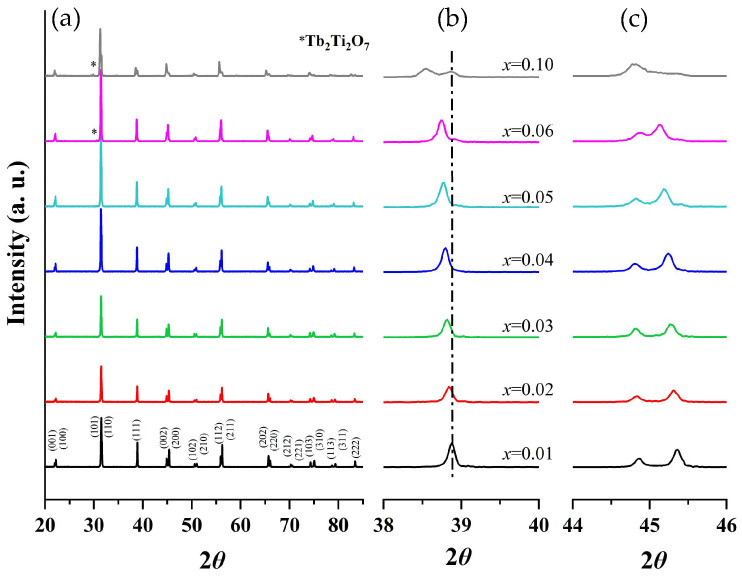
(**a**) Room-temperature XRD patterns of (Ba_1−*x*_Tb*_x_*)(Ti_1−*x*_Ho*_x_*)O_3_ (BTTH) ceramics, and magnified diffraction peaks at (**b**) 39° and (**c**) 45°.

Powder XRD patterns of BTTH ceramics are shown in Figure 1a. When x ≤ 0.05, the samples can be indexed by a single perovskite structure. However, a secondary phase of Tb_2_Ti_2_O_7_ (JCPDS Card No. 15-9785) emerges when x ≥ 0.06, indicating that the solid solubility of Tb/Ho is 0.05. The secondary phase of Tb_2_Ti_2_O_7_ was also reported in Tb-doped barium titanate ceramics with self-compensation of Tb ions [25]. Secondary phases such as Ho_2_O_3_, Ho_2_Ti_2_O_7_, or Ba_6_Ho_2_Ti_4_O_17_ are not observed in all samples within the resolution limit of the XRD equipment, suggesting that Ho ions can completely enter the BaTiO_3_ lattice.

**Figure 2:**
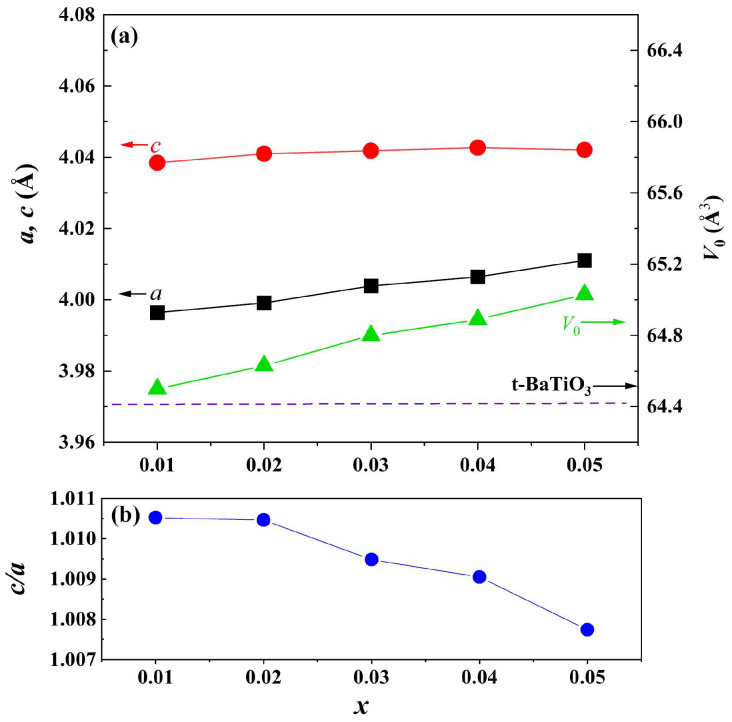
(**a**) Lattice parameters (*a*, *c*) and unit cell volume (*V*_0_) (the dashed line shows the cell volume of tetragonal BaTiO_3_ ceramic) and (**b**) lattice parameter ratio *c*/*a* as a function of doping content.

The magnified diffraction peaks in the vicinity of 39° and 45° are presented in Figure 1b,c. The peak at 39° shifts towards lower diffraction angles as the doping content increases, indicating an expansion of the lattice parameter. Two separate peaks are observed in the region of 44–46 degrees and are assigned to (002) and (200) crystal planes, indicating that the samples possess a tetragonal structure when *x* ≤ 0.06. These two peaks are close together and merge into one peak when *x* = 0.1. To analyze the structural evolution quantitatively, Rietveld refinements for XRD data of samples with pure phase (*x* ≤ 0.05) were performed, and the lattice parameters are shown in Figure 2a. With increasing the doping content, lattice parameter *a* increases from 3.99 to 4.01 Å, whereas *c* remains nearly constant. The unit cell volume (V_0_) also increases almost linearly with doping concentration, obeying Vegard’s law very well [26]. Figure 2b shows the lattice parameter ratio of *c*/*a*, which decreases slightly from 1.011 to 1.008, indicating tetragonality (*c*/*a*) of the crystal structure decreases with doping content, which is consistent with the result of Figure 1.

**Figure 3:**
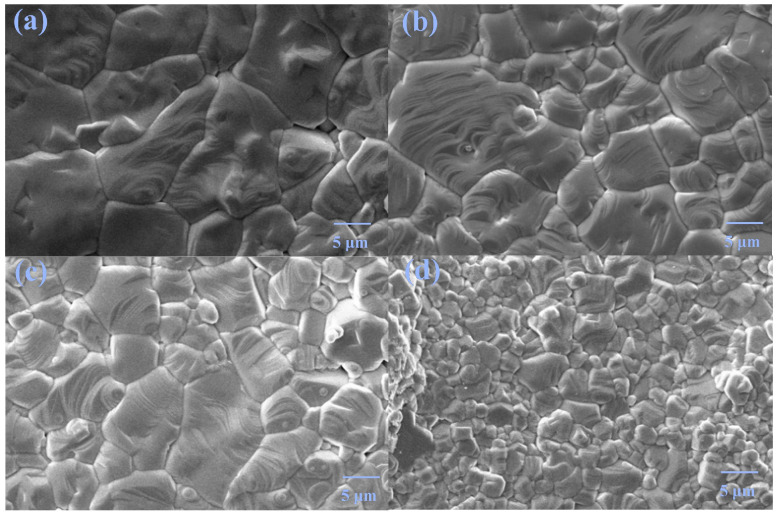
SEM images of *x* = (**a**) 0.02, (**b**) 0.03, (**c**) 0.04, and (**d**) 0.05.

Figure 3 shows the micrographs of pure phase samples. All samples possess good crystallinity with very few pores. The grain size decreases with increasing doping content, suggesting that Tb/Ho co-doping can inhibit the grain growth. Using the Nano Measure software, the grain sizes of the samples are calculated to be 9.17, 6.48, 5.74, and 2.80 μm for *x* = 0.02, 0.03, 0.04, and 0.05, respectively.

### 3.2. Dielectric Properties

**Figure 4:**
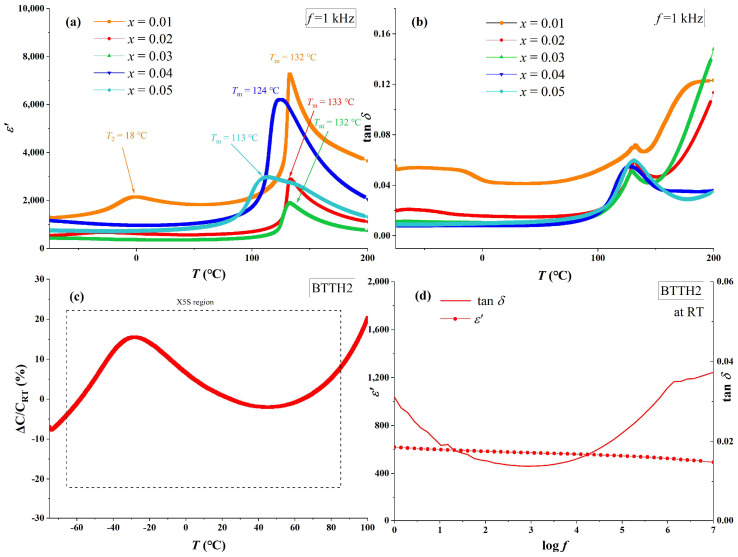
(**a**) Real part of permittivity (*ε*′), (**b**) loss (tan δ) of BTTH ceramics, and (**c**,**d**) dielectric performance of *x* = 0.02.

The temperature-dependent real-part permittivity (*ε*′) and loss (tan *δ*) of BTTH ceramics at 1 kHz are shown in Figure 4a,b. For sample *x* = 0.01, the phase transitions of tetragonal to cubic (*T*_m_) and orthorhombic to tetragonal (*T*_2_) are clearly observed at 132 and 18 °C, which are higher than those of pure BaTiO_3_. This result indicates that a lower doping concentration of Tb/Ho can raise the phase transition temperature, which is also reported by Lu et al. in their study of Tb-doped barium titanate (Ba_1−*x*_Tb*_x_*)(Ti_1−*x*_Tb*_x_*)O_3_ with self-compensation mode [24]. The values of *T*_m_ for *x* = 0.01, 0.02, and 0.03 are almost the same and decrease to 124 and 113 °C for *x* = 0.04 and 0.05, respectively. Sample *x* = 0.01 possesses the highest permittivity among all samples and the sharpest permittivity peak at *T*_m_, which broadens as doping content increases, and the phase transition of orthorhombic to tetragonal (*T*_2_) disappears when *x* ≥ 0.02. Compared with other samples, the loss values for *x* ≥ 0.02 are much lower than that of *x* = 0.01, which is similar to Nd/Yd co-doped BaTiO_3_ ceramics [17]. This result suggests that charges in sample *x* = 0.01 are not compensated and thus lead to higher electrical conductivity.

For sample *x* = 0.02, the dielectric performance satisfies the X5S dielectric specification (−55~85 °C, ǀε′ − ε′_RT_ǀ/ε′_RT_ ≤ 22%). The real part of permittivity and loss at room temperature and 1 kHz for *x* = 0.02 are 617 and 0.015, respectively. In the study of Lu et al., the sample (Ba_1−*x*_Tb*_x_*)(Ti_1−*x*_Tb*_x_*)O_3_ (*x* = 0.05) also satisfies the X5S specification [24]; however, the room temperature permittivity of (Ba_1−*x*_Tb*_x_*)(Ti_1−*x*_Tb*_x_*)O_3_ (*x* = 0.05) is much higher than that of BTTH (*x* = 0.05) samples. This result indicates that the permittivity is significantly influenced when Tb is replaced by Ho. Figure 4d also presents the frequency dependence of permittivity at room temperature for *x* = 0.02. The tanδ values are lower than 0.02 in the frequency range between 10 and 8.8 × 10^4^ Hz, and the values of *ε′* decrease slowly from 621 to 500 when frequency increases from 1 to 10^7^ Hz, suggesting that non-intrinsic polarization can be neglected in the sample [27].

### 3.3. Site Occupation

**Figure 5:**
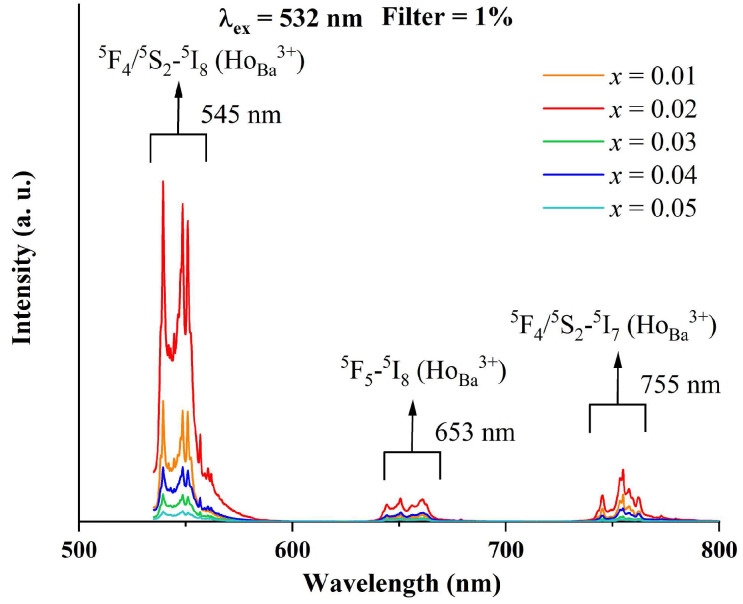
Photoluminescence spectra of BTTH ceramics.

Figure 5 exhibits the photoluminescence spectra of BTTH samples upon the excitation of the 532 nm laser line. There are three PL bands located around 545, 653, and 755 nm with different intensities in the wavelength range of 500~800 nm. Kumar et al. examined the PL spectra of Ho^3+^ in alkali bismuth gallate glasses using excitation wavelengths of 488 and 450 nm [28]. They reported three PL bands: the strong green emission located at 545 nm is assigned to the ^5^F_4_/^5^S_2_ → ^5^I_8_ transition, and the weak red emissions located at 662 and 756 nm are attributed to ^5^F_5_ → ^5^I_8_ and ^5^S_2_ → ^5^I_7_ transitions [28]. Babu et al. reported a similar result of Ho^3+^ as a luminescence center in multicomponent fluorophosphate-based glasses [29]. Battisha and Secu et al. studied Ho-doped BaTiO_3_ ceramics and declared that Ho^3+^ is mainly substituted for the Ba sites under the low sintering temperature [30,31]. They observed four luminescence bands at 435, 545, 660, and 760 nm, respectively, and the last three bands were associated with the ^5^F_4_/^5^S_2_ → ^5^I_8_, ^5^F_5_ → ^5^I_8_ and ^5^F_4_/^5^S_2_ → ^5^I_7_ transitions, respectively. Based on the above discussion, the observed PL spectra of BTTH ceramics in this paper are associated with Ho^3+^ ions at Ba sites. Lu et al. reported three PL bands at 545, 653, and 755 nm and also assigned them to the ^5^F_4_/^5^S_2_ → ^5^I_8_, ^5^F_5_ → ^5^I_8_, and ^5^F_4_/^5^S_2_ → ^5^I_7_ transitions of Ho^3+^ ions on the Ba sites [32]. Makovec et al. investigated the solubility and site occupancy of Ho ions in BaTiO_3_ and drew the conclusion that the solubility limit of Ho^3+^ ions on Ba sites is less than 1.4% at the sintering temperature of 1400 °C in the TiO_2_-rich samples, and Ho^3+^ ions entered the Ti sites in BaO-rich samples [33]. The results of Makovec et al. convincingly proved that Ho^3+^ ions possess an amphoteric behavior of site occupancy, which is influenced by the ratio of Ba/Ti. In Figure 5, the PL intensity of *x* = 0.02 is much higher than that of *x* = 0.01, suggesting a higher concentration of Ho^3+^ at Ba sites with increasing doping content. However, the PL intensity decreases dramatically when *x* > 0.02, suggesting that Ho^3+^ ions at Ba sites are strongly suppressed. Since there is no impurity phase that contains the Ho element in XRD spectra, it is therefore reasonable to suppose that Ho^3+^ ions prefer to occupy Ti sites at higher doping levels.

**Figure 6:**
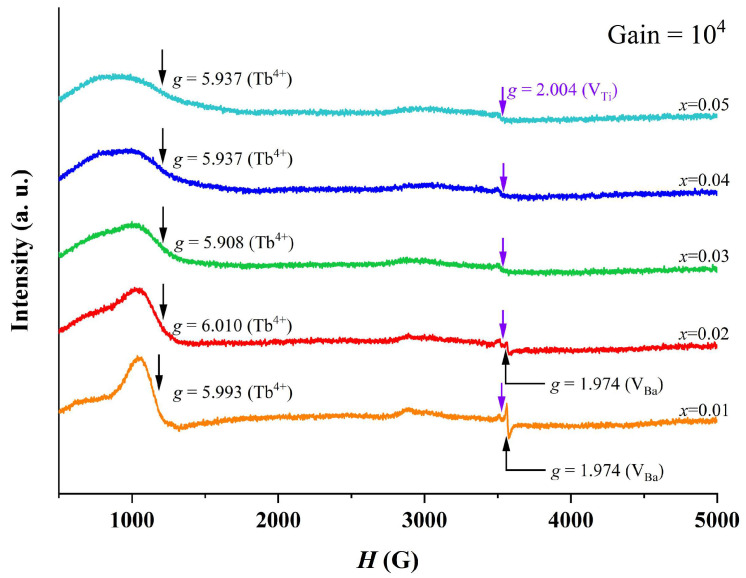
Room temperature EPR spectra of BTTH ceramics.

Figure 6 shows room temperature EPR spectra of BTTH ceramics, and three signals at g = 1.974, 2.004, and 6.010 are observed. The g = 1.974 signal is assigned to ionized Ba vacancy defect ($V_{Ba}^{\prime \prime }$) [34,35], which only exists in *x* = 0.01 and 0.02. For the weak signal, g = 2.004, which is associated with Ti vacancies ($V_{Ti}^{\prime \prime \prime \prime }$) [35], can be observed in all samples. A broad signal is located at g = 5.908~6.010 and is attributed to the electron paramagnetic resonance caused by Tb^4+^ (4f^7^, ^8^S_7/2_) Kramers ions at Ti sites (${Tb}_{Ti}^{\times }$) [25]. The intensity of the Tb^4+^ signal decreases with increasing doping content, suggesting that the concentration of Tb^4+^ ions incorporated into Ti sites decreases with increasing doping level. Because no impurity phase is observed in XRD results for *x* ≤ 0.05, which means that Tb ions completely enter the BaTiO_3_ lattice, the decrease in the Tb^4+^ signal in Figure 6 suggests that more and more Tb ions occupy the Ba sites as Tb^3+^ [12]. In this study, Tb ions are designed to occupy the Ba sites to compensate for the Ti-site Ho ions, and this compensation mechanism will be partly broken when Tb occupies the Ti sites as Tb^4+^. Ba or Ti vacancies will be produced to compensate the Ba-site rare-earth ions. For *x* = 0.01, the quantity of ${Tb}_{Ti}^{\times }$ is larger than other samples; therefore, the largest quantity of Ba vacancies in *x* = 0.01 is reasonable. With the decrease in the Tb^4+^ EPR peak and more Tb^3+^ ions occupying the Ba sites, it is easy to understand the decrease in the Ba vacancy signal. The above results provide solid evidence of multivalent states and amphoteric behavior of site occupancy of Tb ions in BTTH ceramics.

### 3.4. Valence States

The mixed valence states of Tb ions have been widely confirmed in Tb-doped BaTiO_3_ ceramics. Lu et al. studied the self-compensation of Tb ions in BaTiO_3_ and found that the complete self-compensation mode of Tb^3+^ in BaTiO_3_, i.e., ${Tb}_{Ba}^{\cdot }-{Tb}_{Ti}^{\prime }$, could not be formed like Eu^3+^, Dy^3+^, or Ho^3+^ because Tb ions exist in the mixed-valence states of Ba-site Tb^3+^ and Ti-site Tb^4+^ [25]. In their study, Tb ions exist in the forms of Ba-site Tb^3+^ and Ti-site Tb^4+^, and the concentrations of Tb^3+^ and Tb^4+^ are adjustable and deviate from the designed values to maintain the lattice electroneutrality. These phenomena are also observed in other systems with Tb doping [36,37].

**Figure 7:**
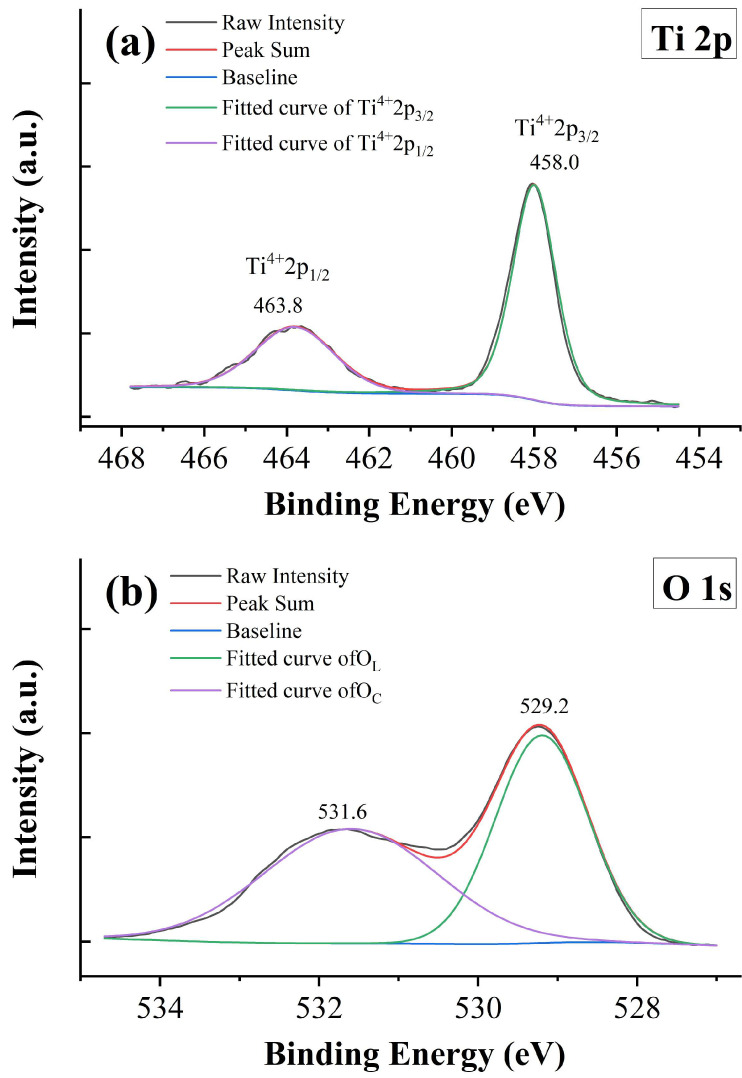

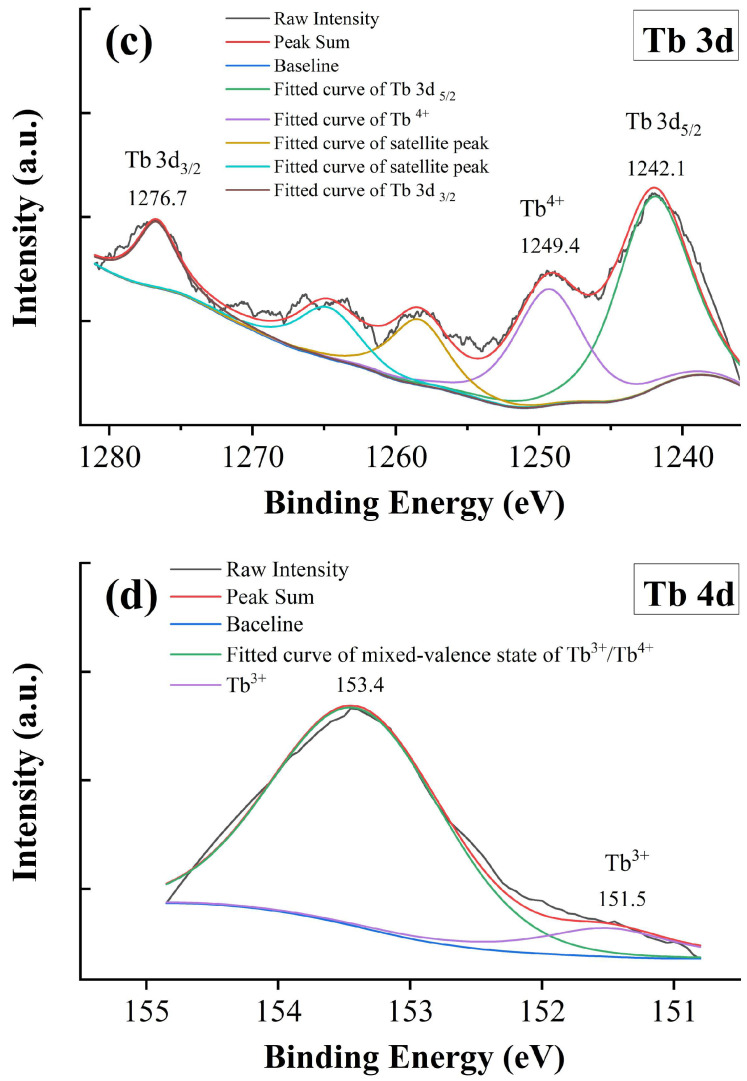
XPS spectra of *x* = 0.05. (**a**) Ti 2*p*, (**b**) O 1*s*, (**c**) Tb 3*d*, and (**d**) Tb 4*d*.

XPS measurement is employed to gain a better understanding of the valences of Tb ions. Figure 7 shows the raw data and fitting results of XPS spectra for sample *x* = 0.05. Figure 7a presents the data for the Ti-2*p* core level, which contains two main peaks originating from the spin-orbit splitting of Ti 2p_1/2_ and Ti 2p_3/2_. The data are fitted with a combination of Gaussian–Lorentzian functions, and only two peaks located at 463.8 and 458.0 eV are obtained. These peaks are identified as a complete Ti^4+^ state, suggesting that the Ti^3+^ state is absent and all Ti ions are tetravalent in the samples. The energy separation of these two peaks is 5.8 eV, which is consistent with the previous studies [38]. It is well known that oxygen vacancies are unavoidable in transition metal oxides under high-temperature sintering conditions and low partial oxygen pressure. For example, Sinclair and West pointed out that La-doped BaTiO_3_ ceramics sintered in an air atmosphere may be oxygen deficient inside the grains [39]. Wang et al. also declared that partial oxidation occurred only in the surface layer of the sample, while the inner part is oxygen deficient [40]. The conducting electrons created by ionization of the oxygen vacancies can be captured by transition metal cations and thus result in the reduction in transition metal cations; for example, Ti^4+^ is reduced to Ti^3+^ [41,42]. However, the mixed valence states of Ti ions do not exist in BTTH. To probe the state of the oxygen anion, the 1*s* core level spectrum of oxygen is also analyzed, and the result is exhibited in Figure 7b. Two peaks are observed at the binding energies of 531.6 and 529.2 eV with an energy separation of 2.4 eV. The XPS spectra of the O 1s core level in BaTiO_3_ or similar systems have been studied a lot [43,44]. According to the literature, the O 1s spectra are usually asymmetric and can be deconvoluted into three peaks, which are associated with lattice oxygen ions in BaTiO_3_ (O_L_), defect oxygen within the BaTiO_3_ matrix (O_D_), and loosely bound oxygen from chemisorbed or dissociated surface oxygen species (O_C_), respectively [43,45]. The energy separation in Figure 7b coincides well with the value of O_C_ with respect to O_L_ [43]; therefore, the XPS spectra of the O 1s core level in this study are assigned to O_L_ and O_C_, and the defect oxygen species is absent, which is consistent with the tetravalent state of Ti ions. According to the literature, defect oxygen is usually associated with the Ti^3+^ state [43,44].

The Tb 3*d* and 4*d* XPS spectra of *x* = 0.05 are shown in Figure 7c,d. Two main peaks are located at 1242.1 and 1276.7 eV, which are associated with the Tb 3*d*_5/2_ and 3*d*_3/2_ signals, respectively. Ramesh and Liang et al. attributed the above signals observed in Tb-doped Zn_2_(BO_3_)(OH)_0.75_F_0.25_ and NaSrB_5_O_9_ phosphor to the +3 state of Tb [46,47]. Colomer et al. reported a mixed-valence state of Tb^3+^/Tb^4+^ in Tb-doped TiO_2_ using the XPS spectrum of the Tb 3*d* region [48]. However, it is still difficult to identify the Tb^3+^ and Tb^4+^ states within the Tb 3*d* region because of their overlapping [12]. Generally, there are satellite peaks with binding energy 10 eV higher than those of the main peaks, whereas no 3*d* satellite of Tb^3+^ exists [49,50]. In this study, the satellite at 1249.4 eV is an indicator of the Tb^4+^ state; however, the Tb^3+^ should not be excluded, especially when the satellite peaks are not significant [49]. Figure 7d shows the XPS spectrum of Tb 4*d*, and the signal within the measurement range can be deconvoluted and fitted with two peaks at 151.5 and 153.4 eV, respectively. The peak at 153.4 eV is related to a mixed-valence state of Tb^3+^/Tb^4+^ [12], whereas the signal at 151.5 eV can be attributed to the Tb^3+^ state [51]. These results provide convincing evidence of the mixed-valence state of Tb ions in BTTH ceramics.

### 3.5. Defect Chemistry

Based on the above results and discussion, the dominant defects in BTTH ceramics are ${Ho}_{Ba}^{\cdot }$, ${Ho}_{Ti}^{\prime }$, and ${Tb}_{Ba}^{\cdot }$. In addition, a small quantity of Ti and Ba vacancies exists in all samples, and *x* ≤ 0.02, respectively. Ho^3+^ prefers to occupy the Ba sites (${Ho}_{Ba}^{\cdot }$) and Tb^4+^ prefers to occupy Ti sites (${Tb}_{Ti}^{\times }$) when *x* ≤ 0.02, whereas Tb^3+^ ions at Ba sites (${Tb}_{Ba}^{\cdot }$) and Ho^3+^ at Ti sites (${Ho}_{Ti}^{\prime }$) will be significant when *x* ≥ 0.03. Therefore, the majority of defects are ${Ho}_{Ba}^{\cdot }$ and ${Tb}_{Ti}^{\times }$ when *x* ≤ 0.02. In this case, Ti and Ba vacancies are produced to preserve the lattice electroneutrality. Thus, the dominant defect complexes in *x* = 0.01 and 0.02 are $2{Ho}_{Ba}^{\cdot }-V_{Ba}^{\prime \prime }$ and $4{Ho}_{Ba}^{\cdot }-V_{Ti}^{\prime \prime \prime \prime }$, which are in agreement with the EPR result. Because the signal of $V_{Ba}^{\prime \prime }$ in EPR is stronger than that of $V_{Ti}^{\prime \prime \prime \prime }$ in *x* = 0.01, the number of $2{Ho}_{Ba}^{\cdot }-V_{Ba}^{\prime \prime }$ should be much larger than that of $4{Ho}_{Ba}^{\cdot }-V_{Ti}^{\prime \prime \prime \prime }$, which means that Ba vacancy is the predominant compensation mechanism for ${Ho}_{Ba}^{\cdot }$. However, due to the largest quantity of ${Tb}_{Ti}^{\times }$ in *x* = 0.01, the quantity of $V_{Ba}^{\prime \prime }$ cannot completely compensate ${Ho}_{Ba}^{\cdot }$, and the extra ${Ho}_{Ba}^{\cdot }$ act as donors, resulting in a high loss value of tanδ. For sample *x* ≥ 0.03, the dominant defects are ${Tb}_{Ba}^{\cdot }$ and ${Ho}_{Ti}^{\prime }$, and they can form the defect complex of ${Tb}_{Ba}^{\cdot }-{Ho}_{Ti}^{\prime }$ without the necessary formation of $V_{Ba}^{\prime \prime }$ or $V_{Ti}^{\prime \prime \prime \prime }$. The vanishing of $V_{Ba}^{\prime \prime }$ signal in the EPR measurement also proves this result. The small amount of $V_{Ti}^{\prime \prime \prime \prime }$ is related to ${Tb}_{Ti}^{\times }$, which cannot be compensated by ${Ho}_{Ba}^{\cdot }$ or ${Tb}_{Ba}^{\cdot }$.

## 4. Conclusions

Single-phase Tb- and Ho-co-doped BaTiO_3_ ceramics with a solubility limit of 0.05 were successfully prepared by the solid-state-reaction method at 1400 °C. The tetragonality and grain sizes of the samples decrease with increasing doping content. The dielectric constant peaks broaden with increasing Ho and Tb content, and the dielectric performance of x = 0.02 satisfies X5S dielectric specification. Both Ho and Tb ions exhibit amphoteric behavior of site occupancy. Tb ions exhibit mixed-valence states of Ba-site Tb^3+^ and Ti-site Tb^4+^, whereas Ho ions are trivalent at both Ba and Ti sites. At low doping levels, Ho and Tb ions prefer to occupy Ba and Ti sites, respectively. For samples with high doping concentration, Ho ions have a tendency to occupy Ti sites, and more Tb ions enter Ba sites. The defect compensation mechanism is also influenced by site occupancy.

## Data Availability

The original contributions presented in this study are included in the article. Further inquiries can be directed to the corresponding author.
